# Relationship between attachment site of tibialis anterior muscle and shape of tibia: anatomical study of cadavers

**DOI:** 10.1186/s13047-022-00559-y

**Published:** 2022-07-12

**Authors:** Kentaro Kimata, Shun Otsuka, Hiroki Yokota, Xiyao Shan, Naoyuki Hatayama, Munekazu Naito

**Affiliations:** 1 Department of Anatomy, Aichi Medical University School of Medicine, 1-1 Yazakokarimata, Nagakute, Aichi Japan; 2 Department of Judo Therapy, Chuwa Professional Training College of Medical Care, 1-1-81 Higashimidori, Inazawa, Aichi Japan; 3 Department of Mechanical Engineering, Meijo University, 1-501 Shiogamaguchi, Tenpaku, Nagoya, Aichi Japan

**Keywords:** Tibialis anterior muscle, Tibia, S-like curve, Cadaver, Sex differences

## Abstract

**Background:**

Tibialis anterior (TA) muscle is the largest dorsiflexor of the ankle joint and plays an important role during gait movement. However, descriptions of the TA attachment site are inconsistent even among major anatomy textbooks, and its origin, especially the attachment site for the tibia, has not been reported in detail. This study is the first experimental attempt to investigate the origin of the TA in detail, paying particular attention to the relationship with the shape of the tibia, including sex differences.

**Methods:**

Forty legs (20 males, 20 females) from twenty Japanese cadavers were examined. Gross anatomical examination of the TA's attachment site to the tibia and the tibia's shape was performed.

**Results:**

The location of the distal end of the TA's attachment on tibia was significantly more distal in males than in females (*p* < 0.01). The anterior border of the tibia had a gentle S-like curve, with a medially convex curve in the proximal region and a laterally convex curve in the distal region in frontal plane. The most protruding point of the distal curve of the anterior border located significantly more proximal in females than in males (*p* = 0.02).

**Conclusions:**

There were sex differences in the distal end of the attachment site on tibia of the TA and the shape of the tibia. Consequently, the variations in the attachment site of TA were considered to provide for differences in function of TA. In males, the TA may enable advantageous power exertion, whereas in females it may work efficiently for dorsiflexion of ankle, respectively. Sex differences in TA's attachment site and the shape of the tibia may be involved in gait movement as well as frequency of lower leg disorders such as chronic exertional compartment syndrome.

**Supplementary Information:**

The online version contains supplementary material available at 10.1186/s13047-022-00559-y.

## Introduction

The tibialis anterior (TA) muscle is the largest muscle in the anterior compartment of the lower leg, accounting for over 60% of the ankle dorsiflexor muscle volume [1, 2]. The TA contributes to the inversion and dorsiflexion of the ankle joint and is involved in maintaining the medial arch of the foot [3]. During locomotion, the TA is active at the heel strike and during swing phase to control foot drop and prevent tripping, respectively [4, 5]. The TA’s activity increases as walking speed increases and decreases when switching to running [4, 6]. The TA is one of the most important muscles in daily life because it is deeply involved in human movement [2, 7].

Several studies have examined the morphological properties of the TA. Wolf and Kim [8] reported that the TA is a pennate muscle composed of three partitions: superficial longitudinal fibers, deep longitudinal fibers, and oblique fibers. The fibers of the TA tendon reportedly rotate approximately 90 degrees from the musculotendinous junction to their insertion on the medial cuneiform and first metatarsal bone [9, 10]. Furthermore, the tendon of the TA has individual variations, such as the number of bands, thicknesses, and insertion sites [10, 11]. On the other hand, several major anatomy textbooks have described that the TA originates from 1/2 to 2/3 of the distance proximal to the lateral surface of the tibia, which is inconsistent with each other [12–14].

Several studies have compared gait movement between men and women and reported that stride length was significantly longer in men, while cadence was significantly greater in women [15–17]. These sex differences in gait are thought to be affected primarily by height and leg length and partially can lead to morphological variations in TA that are involved in ankle dorsiflexion efficiency. It is also important to elucidate the origin of the TA to understand the motor characteristics of the ankle joint during walking motion. However, the anatomical features of the TA that might be different between sexes in gait have not been examined. The anterior border of the tibia is an important attachment site of the TA and appears to have a gentle S-like curve in the frontal plane. As the TA runs along the tibia, this S-like curve influences the attachment pattern and function of the TA. To the best of our knowledge, the origin of the TA, especially the attachment site for the tibia, has not been reported in detail. This study is the first to investigate the origin of the TA in detail for the first time, paying particular attention to the relationship with the shape of the tibia, including sex differences.

## Materials and Methods

Twenty Japanese cadavers (10 male, 10 female) were examined in this study. The ethics committee of Aichi Medical University School of Medicine approved this study (approval no. 2020-M131). The mean ± standard deviation age of the cadavers was 83.2 ± 8.0 years (males, 82.3 ± 5.7 years; females, 84.1 ± 9.7 years). Forty legs (20 males, 20 females) were dissected to assess the attachment site of the TA to the tibia; none showed any apparent signs of deformation or atrophy.

**Fig. 1:**
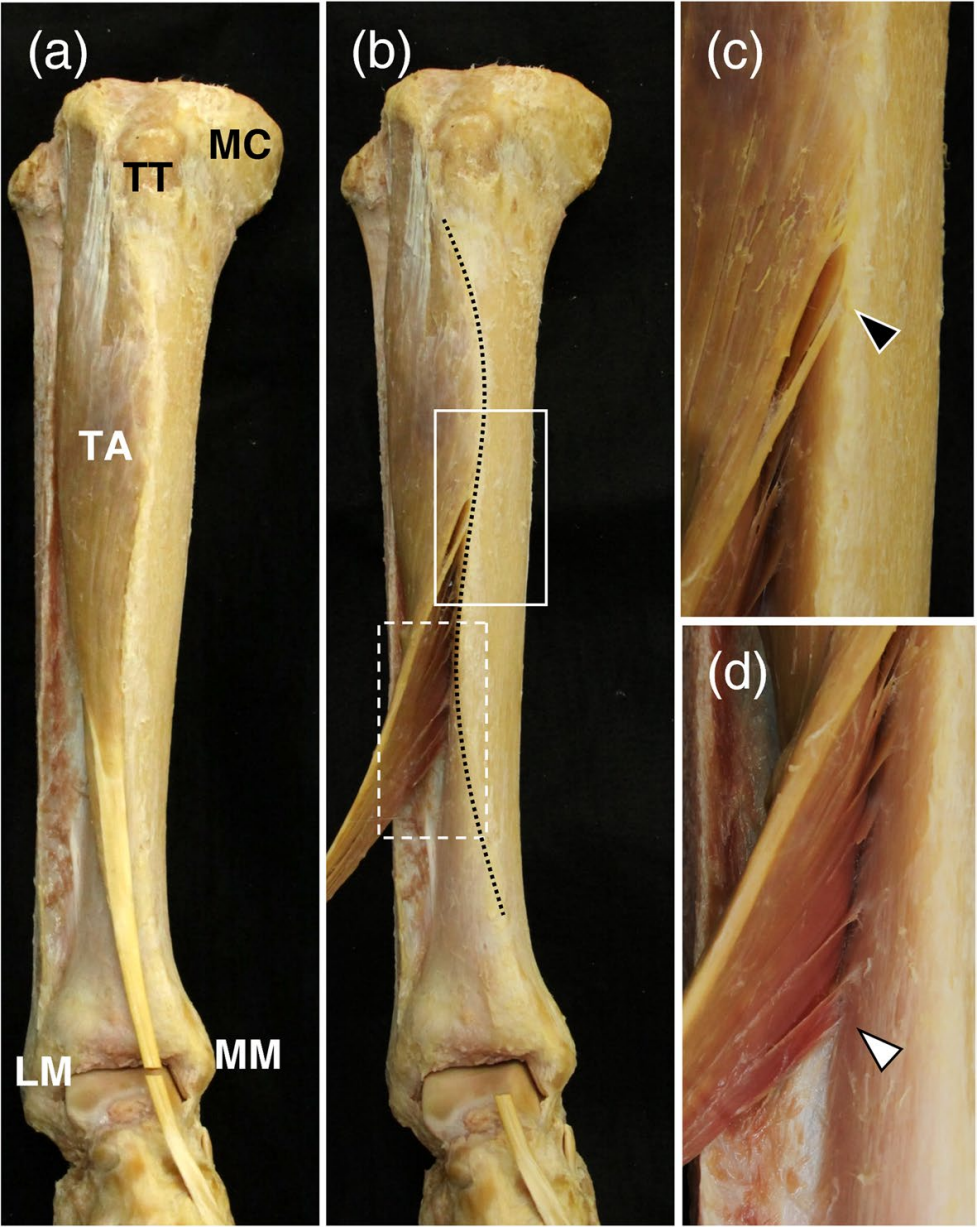
Dissection of the tibialis anterior muscle (TA). (a) Anterior view of the right TA (the tendon was cut at the ankle joint level). (b) The distal ends of the tibial attachment of the anterior and posterior fibers of the TA are shown. The distal part of the TA was turned over. The dotted curve shows the anterior border of the tibia. (c) and (d) Enlarged images of the white solid and dashed squares of (b), respectively. The black and white arrowheads indicate the most distal attachment points of the anterior and posterior fibers of the TA to the tibia. LM, lateral malleolus; MC, medial condyle; MM, medial malleolus; TA, tibialis anterior; TT, tibial tuberosity

**Fig. 2:**
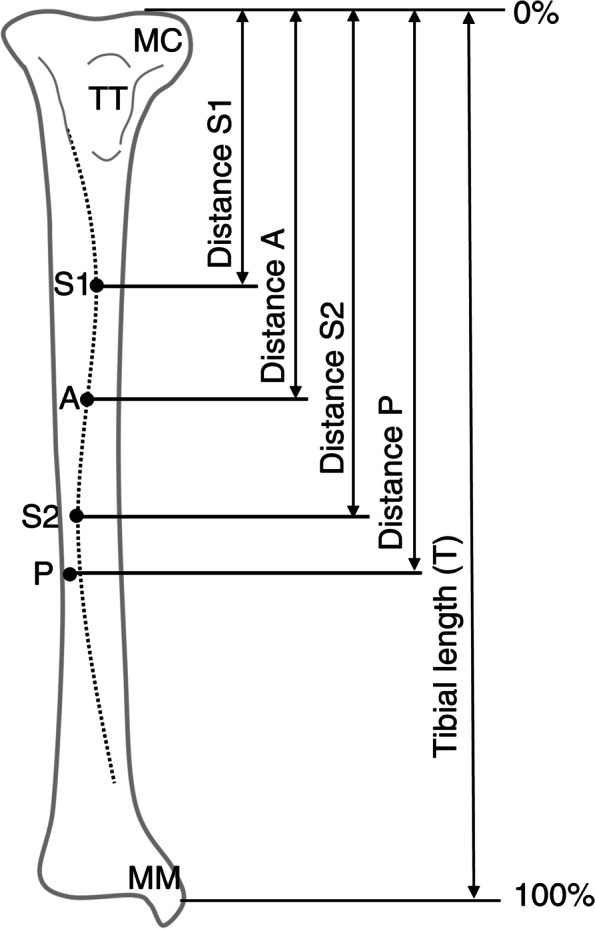
Measurement of the parameters of the TA and the tibia. The tibial length was measured from the upper end of the medial condyle to the top of the medial malleolus. Points A and P indicate the levels of attachment of the anterior and posterior fibers of the TA, respectively. The dotted S-curve shows the anterior border of the tibia. The levels at which the S-curve protrudes most medially and laterally are indicated as S1 and S2, respectively. The length from the upper end of the medial condyle to points A, P, S1, and S2 were measured (distances A, P, S1, and S2, respectively). The ratio of the length from the upper end of the medial condyle to points A, P, S1, and S2 to the tibial length (T) was calculated as a percentage (A-T, P–T, S1-T, and S2-T ratios, respectively). MC, medial condyle; MM, medial malleolus; TT, tibial tuberosity

All dissections and measurements were performed in 2020 by the first author (KK), who has been engaged in musculoskeletal studies for over 10 years. Skin, adipose, and connective tissues were manually removed from the cadavers' legs. Gross anatomical examination of the TA's attachment site to the tibia and the tibia's shape was performed. The TA tendon was cut at the ankle joint level and carefully turned laterally to determine the origin of the tibia. The site where muscle fibers firmly adhered to the bone cortex was defined as the origin of the TA (Figs. 1-a, 1-b). Then, the distal ends of the tibial attachment of the anterior and posterior fibers of the TA were identified (Figs. 1-c, 1-d) and labeled points A and P, respectively (Fig. 2). The anterior and posterior fibers of the TA were defined as the fibers located anterior and posterior to the central aponeurosis itself, respectively. An S-curve of the anterior border of the tibia, with medially convex curve in the proximal region and laterally convex curve in the distal region in frontal plane, was also observed (Fig. 1-b). The levels at which the S-curve protrudes most medially and laterally were labeled points S1 and S2, respectively (Fig. 2). The lower legs were set to maximize the widths of the medial and lateral condyles and the following parameters were measured macroscopically in 1-mm increments: tibial length (distance between the upper end of the medial condyle and the top of the medial malleolus) and the distances between the upper end of the medial condyle and points A, P, S1, and S2 (distance A, P, S1, and S2, respectively). All distances were normalized to tibial length by dividing by the individual tibial length (T) and are shown as percentages (A-T ratio, P–T ratio, S1-T ratio, and S2-T ratio, respectively) (Fig. 2).

### Statistical analysis

The sample size was determined by performing a priori power analysis using G*Power software (version 3.1.9.2) with a large effect size of 0.85 and a power of 0.8. Pearson product-moment correlation was used to investigate the relationships between tibial length and the distances A, P, S1, and S2. Normality of data in all groups was assessed by the Kolmogorov–Smirnov test. Sex and right-left differences in the tibial attachment site of the TA and the shape of the tibia were examined using an independent sample *t*-test and a paired *t*-test, respectively. R 4.0.2 (R Foundation for Statistical Computing, Vienna, Austria) was used for all the statistical analyses. The significance level was set at *p* < 0.05.

## Results

**Fig. 3:**
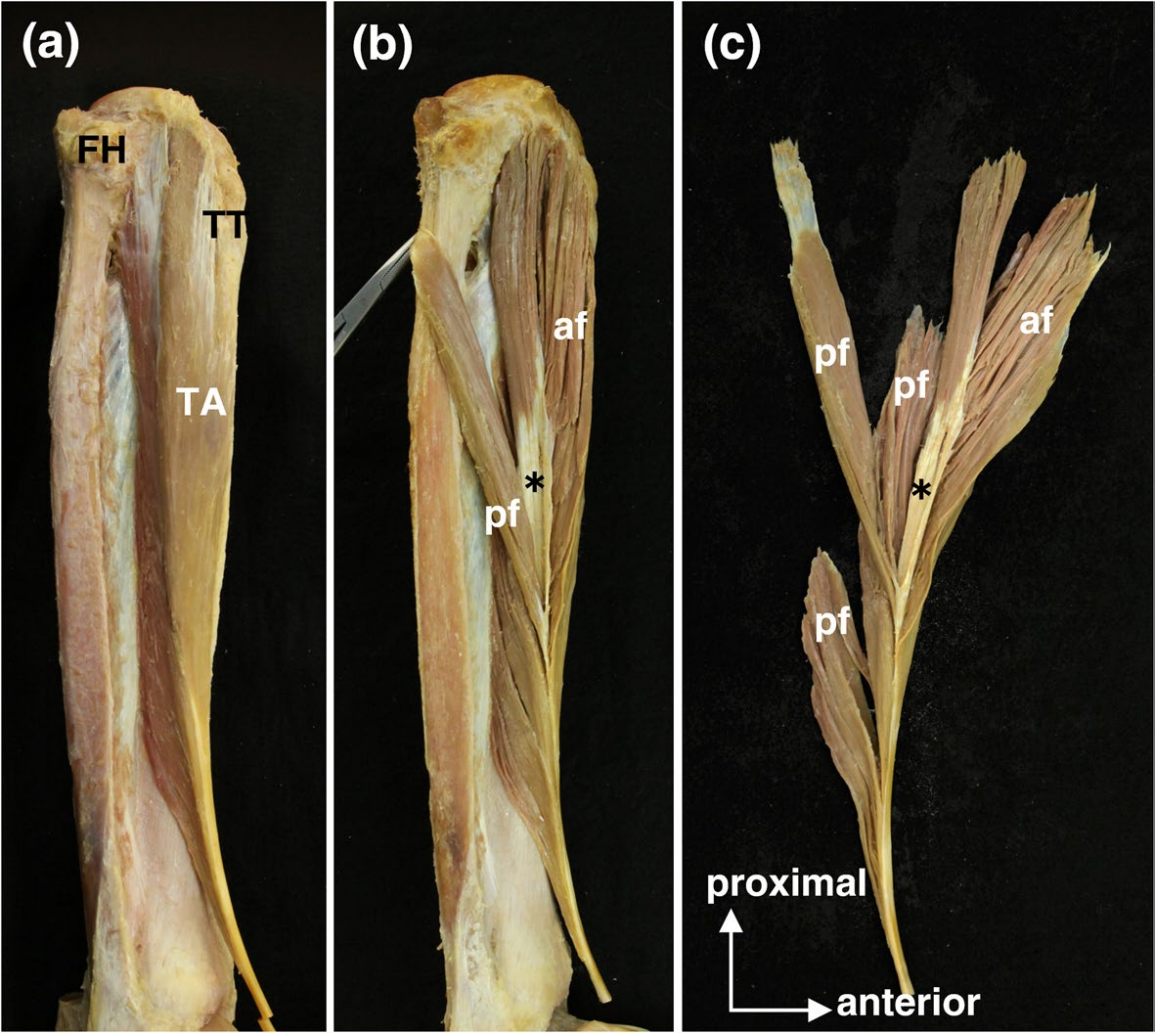
Anterior and posterior fibers of the TA. (a) Lateral view of the right leg. (b) The central aponeurosis was exposed, and parts of the posterior fibers were flipped by forceps. The anterior and posterior fibers were defined by their positional relationship with the central aponeurosis. (c) The TA was removed from the tibia. The anterior and posterior fibers were reflected anteriorly and posteriorly to the central aponeurosis, respectively. af, anterior fiber; FH, fibular head; pf, posterior fiber; TA, tibialis anterior; TT, tibial tuberosity; asterisk, central aponeurosis

The anterior fibers of the TA originated from the lateral condyle and anterior border of the tibia, while the posterior fibers originated from the anterolateral surface of the tibia and the anterior surface of the interosseous membrane. The TA transitioned to a flat tendon while descending the anterolateral surface of the tibia, passed under the extensor retinaculum at the anterior lower end of the lower leg, and then inserted into the medial cuneiform and the first metatarsal bone. The TA was composed of anterior and posterior fibers according to their positional relationship with respect to the central aponeurosis (Fig. 3). The anterior border of the tibia descended from the lateral side of the tibial tuberosity toward the medial malleolus, showing a gentle S-curve. It included a medial convex curve in the proximal region and a lateral convex curve in the distal region of the tibia.

**Fig. 4:**
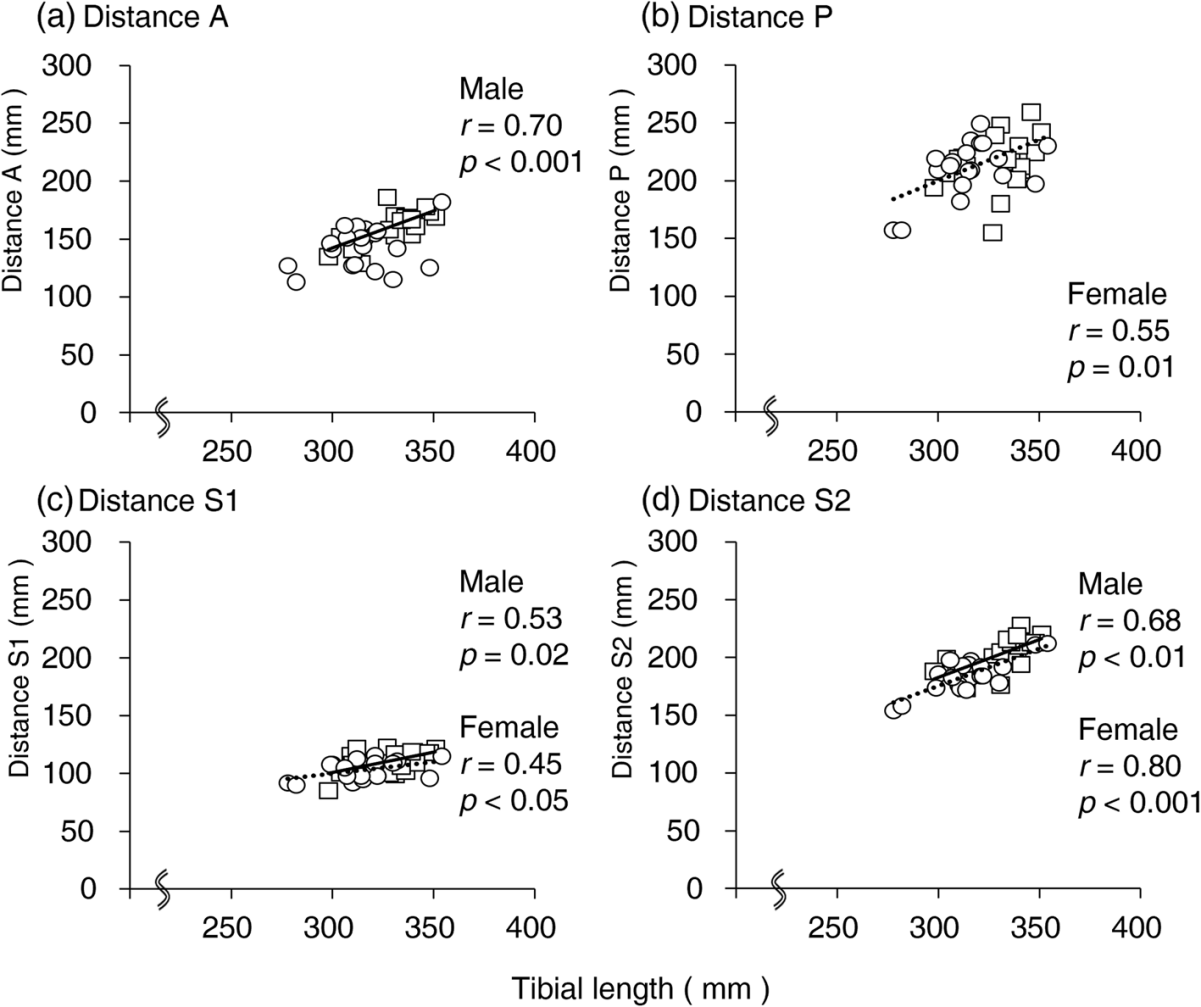
Scatter plots between tibial length and distances A, P, S1, and S2. All values are shown in millimeters. The solid and dotted lines show approximate lines of males and females, respectively. Distance A in females and distance S1 in male were not correlated with tibial length. White squares, male; White circles, female

The anatomical measurements of the TA and tibia are presented in Table 1. In the observed specimens, the tibial length was significantly longer in male (329 ± 16 mm) than in female (315 ± 18 mm) cadavers (*p* = 0.01). Distance A was 152 ± 18 mm (males, 160 ± 14 mm; females, 143 ± 18 mm, *p* < 0.01). There was a positive correlation between distance A and tibial length in males (*r* = 0.70, *p* < 0.001) versus no correlation in females (Fig. 4). The A-T ratio was significantly larger in males (48.7 ± 3.2%) than in females (45.5 ± 5.3%) (*p* = 0.03). Distance P was slightly longer in males (216 ± 23 mm) than in females (210 ± 24 mm). The mean P–T ratio was 66.1 ± 6.4% and showed no significant sex differences (males: 65.6 ± 6.7%, females: 66.7 ± 6.4%). The mean distance S1 of males (111 ± 10 mm) was longer than that of females (103 ± 8 mm) (*p* < 0.01). There was a positive correlation between distance S1 and tibial length in both males (*r* = 0.53, *p* = 0.02) and females (*r* = 0.45, *p* < 0.05) (Fig. 4). The S1-T ratio was not significantly different between males (33.7 ± 2.7%) and females (32.7 ± 2.4%). Distance S2 was longer in males (201 ± 15 mm) than in females (185 ± 15 mm) (*p* < 0.01), while a positive correlation was observed between distance S2 and tibial length in both males (*r* = 0.68, *p* < 0.01) and females (*r* = 0.80, *p* < 0.001) (Fig. 4). The S2-T ratio was significantly smaller in females (58.7% ± 2.9%) than in males (61.2 ± 3.4%) (*p* = 0.02). No significant differences in all parameters were detected between right and left sides.

**Table 1:** Anatomical measurements of the tibia and points A, P, S1, and S2

	Total (*n* = 40)	Male (*n* = 20)	Female (*n* = 20)	Male vs Female		
				Mean difference	95%CI	*p*-value
Tibial length (mm)	322 ± 18	329 ± 16	315 ± 18	14	3, 25	0.01
Distance A (mm)	152 ± 18	160 ± 14	143 ± 18	18	7, 28	0.002
Distance P (mm)	213 ± 23	216 ± 23	210 ± 24	5	-10, 20	0.48
Distance S1 (mm)	107 ± 10	111 ± 10	103 ± 8	8	2, 14	0.007
Distance S2 (mm)	193 ± 17	201 ± 15	185 ± 15	17	7, 26	0.001
A-T ratio (%)	47.1 ± 4.6	48.7 ± 3.2	45.5 ± 5.3	3.2	0.4, 6.1	0.03
P–T ratio (%)	66.2 ± 6.4	65.6 ± 6.7	66.7 ± 6.4	-1.1	-5.4, 3.0	0.57
S1-T ratio (%)	33.2 ± 2.6	33.7 ± 2.7	32.7 ± 2.4	1.0	-0.6, 2.7	0.20
S2-T ratio (%)	59.9 ± 3.3	61.2 ± 3.4	58.7 ± 2.9	2.5	0.5, 4.5	0.02

A and P indicate the distal ends of the tibial attachment of anterior and posterior fibers of the TA, respectively. S1 and S2 indicate the points that the S-curve of the anterior border of the tibia protrudes most medially and laterally, respectively. Values represent means ± SDs. Mean difference = Male – Female; *CI* confidence interval

## Discussion

This study focused on the detailed information of the TA's attachment site to the tibia in addition to the previous studies that have examined the morphology of the TA. This study revealed significant sex difference in the attachment site of TA’s anterior fibers on the tibia, which located more distal in males than females. While there was no significant difference in that of the posterior fibers.

The anterior and posterior fibers of the TA were attached to different levels of the tibia (Fig. 1). In this study, the distal ends of the tibial attachment of the anterior fibers were located more proximally than the posterior fibers. The present study showed that the anterior and posterior fibers of the TA were located at 47.1 ± 4.6% and 66.1 ± 6.4% of the proximal tibia, respectively. Tesch et al. investigated the origin of the TA and its relationship to the tibial shaft by cadaveric lower legs. According to this study, the tibial length was 36.5 ± 3.1 mm and the distal limit of the muscle origin was 12.1 ± 3.3 mm from the tip of the medial malleolus. Therefore, the location was about 66.8% of the proximal tibia, which was similar to that of the posterior fibers of this study. However, the distinction between anterior and posterior fibers of the TA and their sex difference were not examined [18]. According to the major anatomical textbook, the TA arises from the lateral condyle, proximal half to two-thirds of the lateral surface of the tibial shaft, deep surface of the deep fascia, and intermuscular septum between itself and the extensor digitorum longus [13]. The description of “proximal half” in the textbooks corresponds to the distal ends of the attachment of anterior fiber, while that of “two-thirds” corresponds to that of the posterior fiber.

The location that the TA's anterior fibers attach to the tibia was more distal in males than in females, whereas that of the TA's posterior fibers was not different between sexes. Assuming that the length of the tibia and the total length of the TA were constant, extending the muscle attachment area distally increased in the proportion of the muscle belly. Manal et al. investigated the pennation angle of the lower limb muscles using ultrasonography [19]. According to this, male participants had significantly larger pennation angles for the TA at both rest and maximum voluntary contraction compared with the females. The larger pennation angle in males could be explained by the fact that the TA's attachment site extended more distal. These findings are considered to be beneficial for force exertion. Conversely, the anterior fibers of females have relatively longer tendon components than those of males. Tendon length is known as a parameter that increases range of motion, damping, and energy storage, and pennation angle is a factor in determining the force transmitted from muscle fibers to the tendon [20]. The longer tendon component and smaller pennation angle of the TA are considered more advantageous for efficient ankle dorsiflexion. According to previous studies, males and females walked at nearly the same mean preferred speed, while males had longer strides and females had higher cadences [17]. Since females are generally shorter in height and leg length than males and have a disadvantage in stride length, females need to increase cadence relatively. Increasing cadence might lead the TA of female to have an effective function of ankle dorsiflexion, which in turn might lead to morphological changes over time.

Chronic exertional compartment syndrome (CECS) is a common cause of lower leg pain in recreational and competitive athletes [21, 22]. This syndrome is defined as reversible ischemia within a closed fibro-osseous space, which leads to decreased tissue perfusion and ischemic pain [21]. Although there are several reports describing the incidence of CECS, the gender distribution has been inconsistent yet [22–28]. These studies did not anatomically examine sex differences in the affected part. Since CECS is mainly caused by increased internal pressure in the anterior compartment of the lower leg, the anterior fibers of the TA extending more distally in males than in females can be one of the risk factors of CECS. In terms of the variation of muscle attachment site, the current study provides a potential anatomical explanation for the existence of sex differences in risk of developing CECS. The medial tibial stress syndrome (MTSS) is also a common cause of exercise-induced lower leg pain [29]. It was reported that one of the risk factors of MTSS is traction force of muscles applied to posteromedial border of the tibia, the site of symptom [29–31]. It was also reported that females were at greater risk than males [31–33]. Edama et al. focused on the relationship between MTSS and the variation of muscle attachment site [34]. According to this study, it was found that the flexor digitorum longus muscle is closely related to the site of symptoms and that the proportion of the flexor digitorum longs muscle attachment to the site of symptom in females was significantly larger than in males. Anatomical variations of muscle attachment site, including sex differences, may be associated with the development of disorders.

The anterior border of the tibia had a gentle S-curve, with a medially convex curve in the proximal region and a laterally convex curve in the distal region. This is thought to provide a wide area for the TA's origin in the proximal region and play the role of a pulley that regulates the sliding direction of the tendon in the distal portion. In this study, the most protruding point of the distal curve of the anterior border (S2) was located more proximal in females than in males, whereas the most protruding point of the proximal curve (S1) was not different between sexes. The distal end of the tibia is more laterally rotated (tibial torsion) than the proximal end [14]. Furthermore, tibial torsion progresses throughout childhood and adolescence to the point of skeletal maturity, reaching 20–30 degrees in adults [35–37]. It is presumed that the distal part of the S-curve is formed by the tibial torsion and the fact that the tendon of the TA descends medially crossing over the distal tibia. It was also reported that tibial torsion is significantly greater in females than in males [36]. Females have relatively shorter leg lengths but greater tibial torsion than males. This may be the reason female cadavers presented a smaller S2-T ratio than the male cadavers. The proximal S2 distance makes the tendon of the TA closer to vertical and may make ankle dorsiflexion more efficient, especially in females. The TA is known to act as inverter of the foot in addition to dorsiflexor of the ankle [14]. According to previous studies, the moment arm of the TA was found to vary depending upon foot position; the TA exhibited eversion moment arm when the foot was everted and inversion moment arm when the foot was inverted [38]. Wang and Gutierrez-Farewik studied the effect of subtalar inversion/eversion on the dynamic function of the TA, soleus, and gastrocnemius during the stance phase of gait. They found that the TA had large potential to invert the subtalar joint in 1st rocker of stance phase (initial-contact to foot-flat) [39]. Variations in the attachment site of the TA and shape of tibia may affect not only sagittal plane motion but also frontal plane motion, which may be valuable in considering gait patterns.

### Limitation

Although this study has clarified the relationship between attachment site of the TA and shape of tibia for the first time, it has some limitations. In this study, due to the relatively small sample size, statistical analysis could be underpowered. Since the study sample was composed of Japanese cadavers, differences between age groups and racial differences were not considered. The cadavers were embalmed using 10% formaldehyde, hence the measurements could be affected slightly. We did not analyze the strength of the TA and the range of motion of the ankle, because the specimens were from formalin-fixed cadavers. To discuss the dorsiflexion of the ankle, we would like to acquire and analyze these data in the future.

## Conclusion

This study investigated the attachment site of the TA and the shape of the tibia. This revealed that the anterior and posterior fibers of the TA originated from the 1/2 and 2/3 of the distance proximal to the lateral surface of the tibia, respectively. There were sex differences in the relationship between the attachment site of the TA and the shape of the tibia. The variations in the attachment site of TA were considered to provide for differences in function of TA. In males the TA may enable advantageous power exertion, whereas in females it may work efficiently for dorsiflexion of the ankle, respectively.

## Supplementary Information

**Additional file 1:** 

## Data Availability

The dataset used and analyzed during the current study are available from the corresponding author on reasonable request.

## Abbreviations

TA: Tibialis anterior muscle
CECS: Chronic exertional compartment syndrome
MTSS: Medial tibial stress syndrome
