# Supplementary material for: Relationship between attachment site of tibialis anterior muscle and shape of tibia: anatomical study of cadavers
Source: J Foot Ankle Res. 2022 Jul 12;15:54. doi: 10.1186/s13047-022-00559-y (PMC9277928; doi:10.1186/s13047-022-00559-y)
Supplement: Supplementary file 1 — Additional file 1: [file 13047_2022_559_MOESM1_ESM.docx]

July 4, 2022

Prof. Sarah Curran,

*Journal of Foot and Ankle Research*

Ref. No.: JFAR-D-22-00044R2

Title: Relationship between attachment site of tibialis anterior muscle and shape of tibia: Anatomical study of cadavers

We thank you for providing us with the opportunity to resubmit our manuscript titled “Relationship between attachment site of tibialis anterior muscle and shape of tibia: Anatomical study of cadavers” by Kentaro Kimata et al. Furthermore, we would like to express our gratitude to you and the reviewers for your thoughtful insights and comments on our revised manuscript. The manuscript has been rechecked and necessary changes have been made in accordance with all of the reviewers’ comments. We submit herewith a revised version of our paper, with the newly added text highlighted in red.

We have addressed all of the comments by Reviewer #1. Our point-by-point responses to all comments have been prepared and provided below. We hope that our explanations and revisions are satisfactory and that the revised version of our paper is now suitable for publication in the *Journal of Foot and Ankle Research*.

Thank you for your consideration. We look forward to hearing from you at your earliest convenience.

Sincerely,

Shun Otsuka

Department of Anatomy Aichi Medical University

1-1 Yazakokarimata, Nagakute, Aichi 480-1195, Japan

Tel: +81-561-62-3311

E-mail: tsukashun0512@gmail.com

***Response to Reviewer #1:***

***Comment:***

Regarding Table 1:

-please be consistent with the presentation of decimal points for the variable 'mean difference'

-For the following variables, please can you check the 95% intervals, as they do not appear to be symmetrical around their corresponding mean (distance s1, distance s2, distance p, disatnce a, tibial length)

***Response:***

Thank you for your comment. The parameters (tibial length, distance A, distance P, distance S1 and distance S2) were measured in 1-mm increments. Therefore, we have rewritten mean differences and 95% CIs for them after rounded to one decimal place. We have carefully reviewed the 95% CIs and confirmed to be symmetrical around their corresponding mean differences. (page 18, highlighted in red)
